# Revisiting the role of HR in the age of AI: bringing humans and machines closer together in the workplace

**DOI:** 10.3389/frai.2023.1272823

**Published:** 2024-01-15

**Authors:** Ali Fenwick, Gabor Molnar, Piper Frangos

**Affiliations:** ^1^Hult International Business School, Dubai, United Arab Emirates; ^2^The ATLAS Institute, University of Colorado, Boulder, CO, United States; ^3^Hult International Business School, Ashridge, United Kingdom

**Keywords:** HRM, AI, AI-HRM, humanizing AI, human-AI integration, workplace, digital transformation

## Abstract

The functions of human resource management (HRM) have changed radically in the past 20 years due to market and technological forces, becoming more cross-functional and data-driven. In the age of AI, the role of HRM professionals in organizations continues to evolve. Artificial intelligence (AI) is transforming many HRM functions and practices throughout organizations creating system and process efficiencies, performing advanced data analysis, and contributing to the value creation process of the organization. A growing body of evidence highlights the benefits AI brings to the field of HRM. Despite the increased interest in AI-HRM scholarship, focus on human-AI interaction at work and AI-based technologies for HRM is limited and fragmented. Moreover, the lack of human considerations in HRM tech design and deployment can hamper AI digital transformation efforts. This paper provides a contemporary and forward-looking perspective to the strategic and human-centric role HRM plays within organizations as AI becomes more integrated in the workplace. Spanning three distinct phases of AI-HRM integration (technocratic, integrated, and fully-embedded), it examines the technical, human, and ethical challenges at each phase and provides suggestions on how to overcome them using a human-centric approach. Our paper highlights the importance of the evolving role of HRM in the AI-driven organization and provides a roadmap on how to bring humans and machines closer together in the workplace.

## 1 Introduction

The intersection of human resource management (HRM) and technology has always been a dynamic space, constantly adapting to market forces and technological innovations. Over the past two decades, the field of HRM has undergone radical transformations, embracing cross-functionality and data-driven approaches (e.g., Bresciani et al., 2021; Zhang et al., 2021). However, the emergence of Artificial Intelligence (AI) has brought about a paradigm shift in HRM, further altering the role of HRM professionals in organizations. With their capacity for enhancing system efficiency, advanced data analysis, and innovation opportunities, AI technologies have begun to permeate multiple facets of organizational functioning, including human resource management (Guenole and Feinzig, 2018; Rathi, 2018).

Despite the growing interest of AI in both business and HRM scholarship, there is limited understanding on these challenges and the opportunities of AI to improve HRM functions and provide positive outcomes for the wider organization (e.g., Agrawal et al., 2017; Castellacci and Viñas-Bardolet, 2019). Moreover, limited knowledge exists on human-AI interaction at work and how HRM can bring humans and machines closer together (e.g., Arslan et al., 2022). The lack of human considerations in HRM tech design and deployment can hamper AI digital transformation efforts and prevent humans from trusting AI-driven processes and tools (e.g., De Stefano and Wouters, 2022). Our paper addresses this gap in the literature by providing a contemporary and forward-looking perspective to the strategic and human-centric role HRM plays within organizations as AI becomes more integrated in the workplace.

In light of these dynamics, this paper explores the challenges and opportunities presented by AI in HRM. Our primary focus is on the interplay between technology and humanity, and the critical role HRM plays in aligning these forces as AI continues to be integrated in the organization. Using a human-centric approach, our framework provides suggestions on how to overcome existing challenges specifically in people management, culture, and compliance. We provide practical suggestions for addressing existing and future challenges in AI adoption and usage within the field of HRM.

### 1.1 Definitions

HRM is increasingly playing a crucial role in the value creation process of organizations (e.g., DiClaudio, 2019). In this paper we use the definition of HRM by Boselie et al. (2021, p. 484) “*HRM involves management decisions related to policies and practices that together shape the employment relationship and are aimed at achieving certain goals.”* HRM goals can be bundled (Beer et al., 2015) to achieve certain organizational outcomes (such as organizational effectiveness and financial performance) or employee/societal centric outcomes (such as well-being). HRM is often operationalized as a combination of different HRM practices together shaping the various employee relationships that exist in and around the organization (Boselie et al., 2021).

Before addressing the changing role of HRM, we first must define AI because without a clear understanding of the term, it is challenging to discern how HRM practices can effectively harness its potential. Existing definitions (e.g., Afiouni, 2019; Lee et al., 2019; Schmidt et al., 2020; Mikalef and Gupta, 2021) generally converge into two main descriptions: (i) the ability to think, understand, and problem-solve like a human, and (ii) the ability to mimic human thinking. It is also important to clarify the terms “artificial” and “intelligence” when defining AI. “Artificial” typically encompasses anything created by humans (e.g., Simon, 1996; Mikalef and Gupta, 2021). On the other hand, “intelligence” refers to a computer's capability to learn, understand, and reason independently, similar to a human (Russell and Norvig, 2010). Nevertheless, there is currently no widely accepted consensus on precisely defining intelligence (e.g., Wang, 2019). Instead, more philosophical notions of intelligence, such as weak AI and strong AI (Searle, 1980), are often employed to distinguish between varying levels of machine intelligence (Russell and Norvig, 2010). While machine learning (ML) is often used interchangeably with AI, they are not identical. Machine learning is a subset of AI and denotes a set of techniques for solving data-related problems without explicit programming (Kühl et al., 2020). In the context of this paper, we define AI as “*the ability of a machine to learn from experience, adjust to new inputs and perform human-like tasks*” (Duan et al., 2019, p. 63). In this paper the term AI encompasses both rule-based and machine learning techniques (Russell and Norvig, 2010).

Also, there are many definitions of human-centric AI (e.g., Wilkens et al., 2021a,b). Our paper contextualizes human-centric AI as AI tools that prioritize and enhance the human experience by making them more intuitive, empathetic, and aligned with human values and needs. Human-centric AI tools understand and respond to human emotions, enabling natural and empathetic interactions, and respect ethical and social considerations in decision-making processes (Del Giudice et al., 2023). One of the challenges in humanizing AI is that there is no universally accepted approach that guides the best practice for design and use of AI tools. The development of human-centric AI should balance human well-being with technical efficiencies (Bingley et al., 2023). We believe that the concept of humanizing AI should be approached from multiple interconnected perspectives to bridge the existing gaps between humans and machines, which is currently lacking in the field (e.g., Han et al., 2021). In a narrow definition, and in the context of this paper, humanizing AI involves the creation and utilization of AI tools that: (i) enhance human potential, build trust, and minimize fear (ii) can interact with humans in a natural, human-like manner, and (iii) can process information during these interactions in a manner similar to human cognitive processes (Fenwick and Molnar, 2022). AI evolves over a path of maturity spanning a continuum of contemporary cognitive architectures to more socio-cognitive and cross-domain architectures (e.g., Gupta et al., 2023), and in terms of implementation and human-centricity, needs to be interpreted in the context of place and time (Wilkens et al., 2021a). These advancements can help create AI with more general intelligence and support ongoing efforts to bring humans and machines closer together.

### 1.2 The evolving role of HRM; a historical overview

It is important to review the evolution of HRM to better understand how the functions, practices, and philosophies within the field change with time to align with management practices and technological developments, and to identify effective HRM practices in an ever-evolving business environment. Identifying the evolving role HRM has played in humanizing the workplace is equally important.

In the evolution of HRM, existing literature identifies four different stages: administrative HR, personnel management, strategic HR, and business partner HRM (e.g., Fombrun et al., 1984; Kaufman, 2007; Wright, 2008; Kim et al., 2021). Administrative HR is the organization's earliest phase of human resource practices. During this stage, which was most relevant in the early to mid-20th century, HRM primarily focused on administrative and transactional tasks related to compliance and managing the workforce, using paper-based tools, such as manning tables (Mahoney and Deckop, 1986; Hendrickson, 2003). Administrative HR's focus on humanizing the workplace was mainly concentrated on industrial psychology practices for identifying and selecting new hires and other human factor related activities (Münsterberg, 1998). Personnel Management, which gained prominence in the mid-20th century, marked a transition toward a more employee-oriented approach. In this stage, the primary focus shifted from administrative tasks to effectively managing the workforce as an asset. In this stage, various technology tools, such as applicant tracking systems and learning management systems gained popularity to support recruitment and training processes, enhancing employee skills in a more systematic and efficient manner (Kaufman, 2007; Kim et al., 2021). The tenets of humanizing the workplace in this era were based on a behavioral model, emphasizing the importance of understanding how environmental, social, and psychological factors motivate employee behavior and thus productivity. This gave rise to HR practices such as training and development, employee compensation, and communication (e.g., Kaufman, 2015; Armstrong and Taylor, 2020). Strategic HRM emerged as a transformative stage in the evolution of HRM practices to deal with external pressures such as globalization and technological developments, particularly from the late 20th century onwards. It signified a fundamental shift in HR's role within organizations, evolving from a primarily administrative and personnel-focused function to a proactive and strategic partner role integral to achieving organizational goals (e.g., Kaufman, 2007; Kim et al., 2021). The term HRM originated in this time to encompass its multi-faceted nature. During this era, with the emergence of computers and enterprise resource planning (ERP) systems, human resource information systems (HRIS) were used to store and analyze data to increase workflow efficiencies and make data-driven decisions (Hendrickson, 2003). Humanizing the workplace in the strategic HRM phase focuses mainly on enhancing the employer—employee relationship through improved HRM practices and systems for performance management and career planning leading to higher work satisfaction and productivity (Wright, 2008; Kim et al., 2021). Business partner HRM represents the latest evolution in HR practices. In the business partner HRM era, with the rise of the internet at the turn of the century, there is a heightened focus on digital approaches (e-HRM, online HRM, digital HRM) to make more data-informed decisions and create value for the organization (Wright, 2008; Malik et al., 2020). Seeing employees and talent management as a significant source of competitive advantage, enhancing the human experience at work through technology and people-centric approaches like diversity and inclusion become equally important. In this phase, HRM also recognizes the importance of designing and using technology solutions that align with human values and needs (Malik et al., 2020).

With the advent of AI, firms are assessing how they can implement AI technology to enhance efficiency and productivity (Chui et al., 2023). Humanizing the workplace in the digital HRM phase requires an emphasis on using technology to make the organization more human-centric and enhance human values and potential, which, at times, is contrary to efficiency and productivity goals. The AI-driven phase of business partner HRM is a significant turning point in its evolution. Most organizations are unclear on utilizing AI technologies to achieve their people-management and value enhancement goals, raising concerns about AI ethics, compliance, and culture to create a human-centric workplace (Budhwar et al., 2023).

### 1.3 The role of HRM in the age of AI

Despite a long history of enhancing physical abilities and basic cognitive skills, technology has never been able to augment human intelligence at the workplace and beyond. This limitation is changing now. For the first time, technology is enabling the enhancement of human intelligence (Abbass, 2019) and this creates new challenges for HRM. Advanced digital technologies (such as AI including cutting edge machine learning techniques) transforming many HRM functions and practices further enhancing HRM across a range of activities and departments to enhance operational performance and value creation (Dwivedi et al., 2021). Despite the range of benefits and opportunities AI presents to organizations, the challenges of effectively integrating AI technology into HRM are complex (Tambe et al., 2019; Palos-Sánchez et al., 2022). Moving forward, it is important to review these challenges in a systematic way to overcome these complexities. We therefore provide a structured framework, grouping HRM practices into three specific bundles: people management, culture, and compliance. People-related functions encompass talent acquisition, development, and management, focusing on the workforce's growth and well-being. Compliance-related functions revolve around adhering to legal and ethical standards, ensuring organizations operate within regulatory boundaries, and maintaining fairness and equity. Culture-related functions concentrate on shaping organizational culture, fostering collaboration, and promoting values and behaviors that align with the firm's mission. By categorizing HRM practices into these three groups, we align with the primary domains where HRM professionals exert their influence (e.g., O'Donovan, 2019; Johnson et al., 2022; Ammirato et al., 2023; Prikshat et al., 2023). This categorization provides a comprehensive view of HRM's role in addressing diverse organizational needs, from nurturing human capital to upholding ethics, meeting regulations, and nurturing a cohesive workplace culture. It also emphasizes that HRM is not solely about administration; it is a strategic business partner that influences people, culture, and compliance to drive the organization's success (Sakka et al., 2022). Furthermore, our recommended framework highlights the need for a multi-disciplinary approach to HRM that considers the technical, ethical, and human elements within each category. In the next section, we explore how HRM can play a pivotal role in bridging the gap between humans and machines in the workplace.

## 2 How HRM can bring humans and machines closer together in the workplace

The adoption of AI within the field of HRM depends on various technological, business, and human factors. Market demands also impact the decision to use AI within HRM design (e.g., Dwivedi et al., 2021; Nguyen et al., 2022). These factors have varying degrees of development, which can propel or constrain AI implementation within the field of HRM. Moreover, the digitization of HRM (including access to quality and unbiased data) also needs to be carefully managed to mitigate risks and ensure alignment with other business functions (e.g., Malik et al., 2022). It is, therefore, important to review AI design and implementation from a trajectory perspective.

In terms of humanizing AI in the workplace, the function of HRM plays a pivotal and varying role in the process of making AI technical solutions in the workplace more human-centric. The aim is to bring humans and machines closer together. Not taking a human-centric approach to AI usage within HRM not only prevents digital transformation efforts and more data-driven decision-making but also jeopardizes more sustainable human resource management in the digital age (e.g., Budhwar et al., 2022) and further advancement toward safe artificial general intelligence (e.g., Everitt, 2019). Recruitment bias, fear of job loss (Frick et al., 2021; Jöhnk et al., 2021; Uren and Edwards, 2023), ineffective human-machine integration (Arslan et al., 2022), human trust in machines (Gillespie et al., 2021), and concerns of privacy (Bodie, 2022) are some of the most common challenges HRM is facing with AI today and will continue to face moving into the future. Addressing the key challenges at each stage of design and implementation not only helps HRM to reposition itself and the value that it helps create for the organization, but also informs AI development and identifies ways to enhance human properties through emerging technologies.

**Figure 1:**
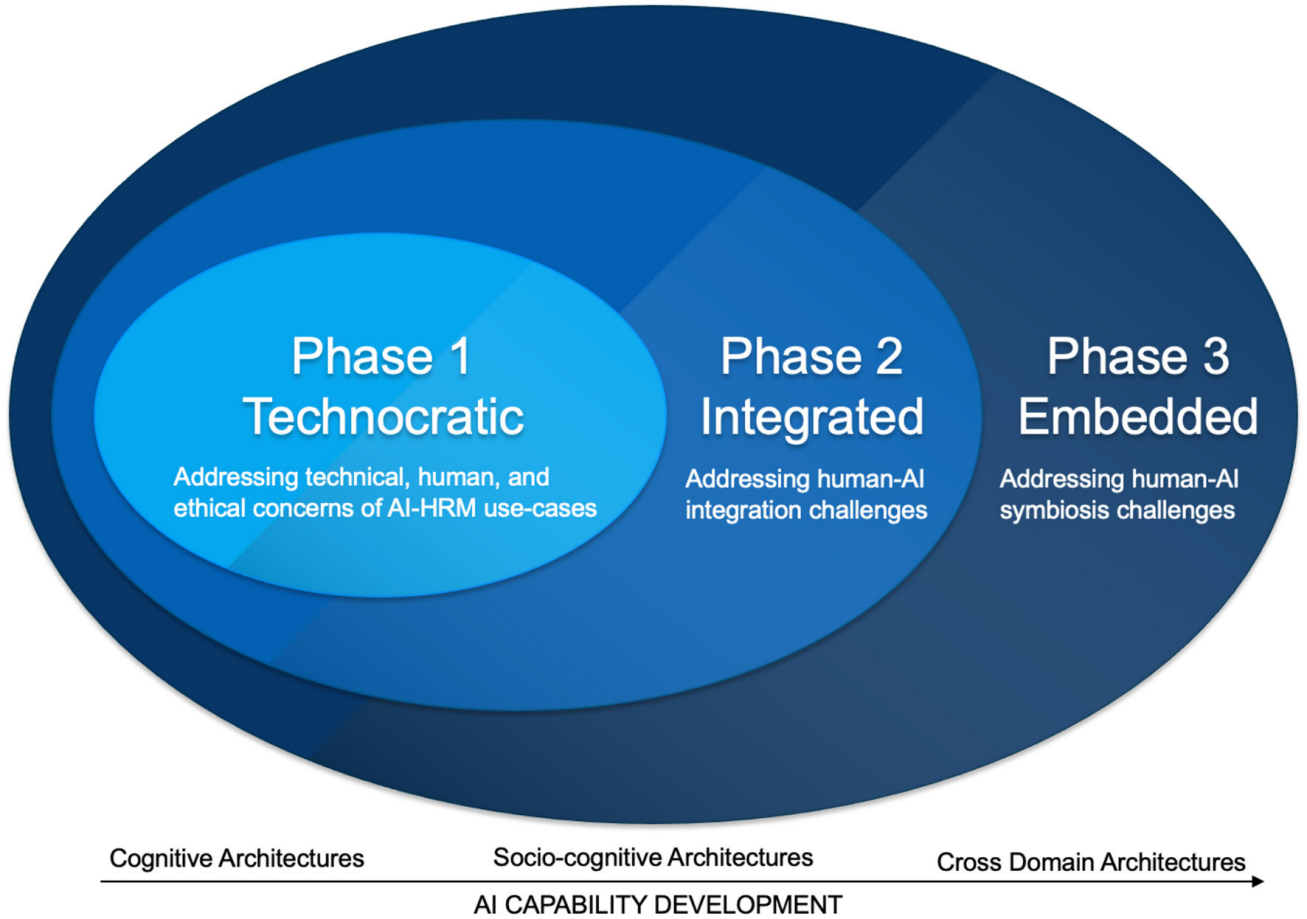
The role of HRM in humanizing AI in the workplace.

Drawing insights from literature on technology adaptation within HRM (e.g., Kim et al., 2021), and the future outlook of AI technology (Kurzweil, 2005; Abbass, 2019; Silichev et al., 2019; He et al., 2021), the following subsections discuss three phases of AI usage in the workplace: (1) technocratic, (2) integrated, and (3) fully embedded, specifically for people management, culture, and compliance, the challenges faced at each stage in terms of humanizing AI, and which opportunities HRM can capitalize on (Figure 1). The technocratic phase represents an initial stage of AI-HRM integration, where AI is primarily used to automate and enhance specific HRM functions and practices. It is characterized by the application of AI in tasks such as HR planning, recruitment, training, and performance management. The integrated phase represents a more advanced stage where AI and humans work more closely together. It involves integrating AI into daily functions, personalizing employee experiences, and emphasizing collaboration between humans and machines. The fully-embedded phase reflects a more mature and evolved stage of AI adoption, where HRM focuses on managing the interaction between humans and AI in a way that enhances the overall human experience and seeks to create a workplace that reflects the broader societal goal of leveraging technology for the betterment of individuals and communities. These three phases, from technocratic to fully-embedded, are derived based on the evolution of AI technology adoption within the field of HRM. The first two phases are based on recent empirical literature on AI in HRM (e.g., Arslan et al., 2022; Bansal et al., 2023; Bujold et al., 2023). The last phase is our conceptual view, and it represents a logical progression of how AI is integrated into HRM practices and aligns with broader developments in technology adoption and societal goals (e.g., He et al., 2021).

### 2.1 AI-HRM human-centric orientation: technocratic phase

Modern technologies, such as AI, machine learning, and AR/VR, play an increasingly vital role within the field of HRM supporting and shaping various people management functions and practices (e.g., Bersin and Chamorro-Premuzic, 2019; Malik et al., 2022). Currently, AI-based applications support HRM professionals with HR planning (e.g., Karatop et al., 2015), selection and recruitment (e.g., Torres and Mejia, 2017; Van Esch et al., 2019), training and development (e.g., Sitzmann and Weinhardt, 2019), performance management (e.g., Bankar and Shukla, 2023), influence employee attitudes such as engagement and work satisfaction (e.g., Castellacci and Viñas-Bardolet, 2019), and support employee retention (e.g., Chowdhury et al., 2023b). AI currently supports and provides HRM functions with various benefits ranging from automating mundane tasks and reducing HR-related costs to debiasing hiring processes and leveraging people analytics to make data-driven decisions (e.g., Henkel et al., 2020).

#### 2.1.1 Challenges

Despite obvious efficiency gains AI brings to organizations, human resource departments are facing new pressures associated with balancing these efficiencies and harmonizing human workforces. AI remains a significant source of concern for employees in many organizations (Palos-Sánchez et al., 2022). Recruitment bias, fear of job loss (Frick et al., 2021; Jöhnk et al., 2021; Uren and Edwards, 2023), ineffective human-machine integration (Arslan et al., 2022), human trust in machines (e.g., Gillespie et al., 2021; Budhwar et al., 2022), managers incomplete understanding of AI systems and their impact on employee outcomes (e.g., Castellacci and Viñas-Bardolet, 2019), existing AI regulatory frameworks too broad to address nuances of AI usage within the context of employment (Chowdhury et al., 2023a), data privacy (Bodie, 2022), and lack of human consideration in AI decision-making (Mazarakis et al., 2023) are some of the most common challenges HRM is navigating with AI.

#### 2.1.2 Opportunities for HRM

The critical role HRM can play in making AI-usage more human-centric is first by providing training and development opportunities to decision-makers in the organization on how AI works and how to use AI in a way that benefits employee and organizational outcomes (e.g., Arslan et al., 2022; Malik et al., 2022). Second, to address the issues of trust in AI, HRM professionals can play a more active role in addressing concerns about job transformation (Huang et al., 2019), professional identity (e.g., Mirbabaie et al., 2022), AI training (Chowdhury et al., 2023a), and have employees be part of the AI implementation decision-making (e.g., Bankins, 2021; Bankins et al., 2022). Alleviating fears and concerns of employees is critical for AI implementation to succeed in the workplace and to identify more effective ways to implement AI in later stages (e.g., Park et al., 2021). Each of these concerns also affects organizational culture. As more and more machines enter the workforce, replacing human beings, questions are emerging on the changing cultural dynamics within firms (Frangos, 2022; RoŽman et al., 2022; Chowdhury et al., 2023a). In the technocratic stage of AI-HRM implementation and usage it is important to develop and nurture an organizational culture of innovation (Fountaine et al., 2019; Pumplun et al., 2019; Ransbotham et al., 2021), collaboration (Fountaine et al., 2019), and effective change management (Pumplun et al., 2019). From a compliance perspective, firms must start with developing their AI policy to comply with the current high-level guidelines of human-centric AI regulations (e.g., de Laat, 2021). AI policies serve as a critical foundation to support AI implementation and usage within the organization, maintain ethical standards, and develop trust with internal and external stakeholders (Sjödin et al., 2021). Finally, HRM can also work as an interface between developers and employees to help address the lack of human consideration when AI makes critical decisions about hiring, firing, and reward allocation (e.g., Malik et al., 2023).

### 2.2 AI-HRM human-centric orientation: human-AI integration phase

Human-AI integration can happen to varying degrees. To date, most human-AI integration focuses on the co-existence of humans together with AI, where humans and AI perform as separate entities. Recent AI developments focus more on human-AI integration, where humans and machines make decisions together (e.g., Einola and Khoreva, 2023). This is often referred to as human-in-the-loop (HITL) (e.g., Monarch and Munro, 2021). In phase two, HRM practices focus on bringing humans and machines closer together by integrating AI more into daily functions of employees (e.g., Rydén and El Sawy, 2022), personalizing employee experiences and learning journeys (e.g., Bulut and Özlem, 2023), and identifying and leveraging human-AI interaction mechanisms in the workplace (e.g., Budhwar et al., 2022; Herrmann and Pfeiffer, 2023). When we look at empirical survey data, high AI performer firms, defined as “*organizations that attribute at least 20 percent of their EBIT to AI adoption*” (Chui et al., 2023, p. 8), already distinguish themselves by integrating AI deeply into their operations, leveraging it not just for cost reduction but to enhance HRM functions and organization design. This comprehensive use of AI in enhancing organizational design and creating new value propositions sets high AI performer firms apart, demonstrating a more integrated and strategic application of AI within their organizations (Chui et al., 2023). As human and machine systems and processes become more integrated in phase two, organizational culture management will evolve as well. Leadership style shifts are most likely to occur as a result of changing employee dynamics influenced by AI implementation (Peifer et al., 2022). In phase two, firms move beyond high-level regulations to anticipate and implement more prescriptive guidelines and controls. This phase will be characterized by meeting not only current regulations but preparing for future regulations designed to address AI's unique challenges (e.g., Hadfield and Clark, 2023). Compliance also plays a stronger role in responsible human-computer interaction (HCI) design and human-computer responsibilities and liabilities (e.g., Rakova et al., 2021).

#### 2.2.1 Challenges

Human-AI integration phase faces unique challenges. Some of the challenges HRM will face in the integration phase are role and job design challenges (e.g., Sampson, 2021), HCI design challenges (e.g., Arslan et al., 2022), human and AI cross-functional team issues (e.g., Klien et al., 2004; Arslan et al., 2022), responsible design (e.g., Bankins, 2021), ethical concerns in terms of decision-making (e.g., Flathmann et al., 2021), cultural differences (Herrmann and Pfeiffer, 2023), and appropriate oversight and governance (e.g., Wu et al., 2020). The main challenges HRM faces in phase two are centered around employee up-skilling and re-skilling, AI solution design and integration challenges, and delineation of responsibility between humans and machines.

#### 2.2.2 Opportunities for HRM

To help address these issues, HRM professionals first can focus training efforts on augmenting existing skills using AI tools and applications so that employees feel more comfortable working with AI technology and making decisions together (e.g., Arslan et al., 2022). Second, HRM continues to work with AI application developers to make sure integrated AI usage is user-friendly, intuitive, explainable, and responsible. Third, study the human-AI interactive mechanisms that amplify human skills and develop guidelines for human-AI collaboration and integration (e.g., Budhwar et al., 2022; Berretta et al., 2023; Hu and Wu, 2023). These efforts to take a human-centric approach to learning and development can motivate employees to learn how to work with new technologies and be more willing to transform with the organization (e.g., Beichter and Kaiser, 2023). Integrated AI tools can also augment human capabilities through a HITL approach in which humans participate in the algorithmic decision-making process, improving the explainability of decision outcomes and human acceptance of algorithm-based decisions (Mosqueira-Rey et al., 2023). As technology advances and moves more into socio-cognitive architecture models, more advanced HITL setups will emerge (e.g., Gupta et al., 2023; Mosqueira-Rey et al., 2023). Finally, anticipating ongoing changes to regulation, including but not limited to anticipated compliance verification requirements, organizations at this stage stay committed to building continuous learning and adaptation mechanisms to minimize liabilities and unethical AI usage in the workplace (e.g., Kulkarni et al., 2021; Wiehler, 2022; Grabowicz et al., 2023; Hu and Wu, 2023).

### 2.3 AI-HRM human-centric orientation: fully-embedded AI phase

The advancement of new AI architectures (moving more toward cross-domain intelligence) and human-computer interaction, together with operationalizing human-AI collaboration in the workplace, starts a new phase in the AI-driven organization. In the fully-embedded phase, AI is more intelligent and less artificial, becoming an imperative within organizations for creating and capturing value. Once the AI-driven organization is fully operational and traditional HRM functions and practices are automated, the role of HRM focuses less on integration and emphasizes more on employee experience and organizational effectiveness, ensuring that they are in line with human-centric principles and ethical standards (e.g., Seidl, 2022). In the fully AI-embedded phase, the functions and processes of HRM are very different than in previous stages. The function of HRM becomes more strategic and human-centered and will focus more on managing organizational and algorithmic behavior to help the organization meet rapidly changing needs (e.g., Langer and König, 2023; Rodgers et al., 2023). The role of HRM includes the management of human resources and technology together due to its increased symbiotic relationship. In the fully-embedded AI phase, HRM becomes an even more multi-disciplinary function, working together with behavioral data scientists, psychologists, and technologists (Fenwick and Molnar, 2022), we therefore propose HRM to reposition itself to Human Technology Resource Management (HTRM).

#### 2.3.1 Challenges

Technology and human resources are both equally important, and the challenge for HRM is to build (or, keep building) a symbiotic relationship between humans and machines. Besides the ongoing focus for re-skilling and job design, challenges could be employee resistance to fully automated AI-HRM (e.g., Brock and von Wangenheim, 2019; Frick et al., 2021), bias and fairness checks (e.g., Zhuo et al., 2023), maintaining human-centricity and purpose-driven approaches (e.g., Cappelli and Rogovsky, 2023), and complex human issues and well-being, such as digital divide and mental health issues (e.g., Khogali and Mekid, 2023). Most of the challenges in phase three center around human well-being, performance optimization, exception handling, and ethics. Increased automation is known to lead to more stress and anxiety in the workplace amongst other psycho-social risks (e.g., Cefaliello, 2021). As AI-powered tools and processes become more “intelligent,” human employees can fear AI and harbor job insecurities and unfair treatment.

#### 2.3.2 Opportunities for HRM

HRM could address these issues from a human-centric approach by ensuring humans are put at the center of AI-HRM development (e.g., Mazarakis et al., 2023). Looking ahead to industry 5.0 (e.g., Coelho et al., 2023), there is a greater focus on the human aspect within organizations aiming to find more sustainable and resilient ways to bring humans and machines together thus rethinking how value is created in today's world (e.g., Del Giudice et al., 2023; Pizoń and Gola, 2023). In phase three, new perspectives of human-AI integration at work are extending to neural integration, where AI tools are embedded into humans (e.g., mind-controlled machines, neurolinks, intelligent prostheses) to enhance human capabilities or human cells are used in bioengineering for the development of organoid intelligence (e.g., Morales Pantoja et al., 2023). With the emergence of advanced integrated human-AI tools and interfaces, we predict that HRM will continue to focus on developing strict adherence to ethical rules (e.g., Pflanzer et al., 2023). The HRM community will also influence regulators to enforce more human-centric policies. Emphasizing the importance of culture in mitigating employee resistance remains a pressing concern for HRM in the future (Ransbotham et al., 2021), as is addressing issues concerning centralized power with the AI-embedded organization (e.g., Einola and Khoreva, 2023). This approach not only fosters ethical AI but also distinguishes organizations as stewards of technology that enhances, rather than diminishes, the human experience.

## 3 Conclusion

In the age of AI, the role of HRM professionals in organizations continues to evolve. AI technologies are increasingly being implemented in organizations to enhance HRM across a range of activities and departments to support operational performance and value creation. A growing body of evidence highlights the benefits AI brings to the field of HRM. Despite the growing interest in AI-HRM scholarship, the focus on human-AI interaction at work and AI-based technologies for HRM is limited and fragmented. Moreover, the lack of human considerations in HRM tech design and deployment can hamper AI digital transformation efforts and jeopardize more sustainable human resource practices in the digital age and even advancements toward safe artificial general intelligence. To provide a structured framework for reviewing these challenges, and based on existing literature (e.g., Ammirato et al., 2023; Prikshat et al., 2023), we grouped HRM practices into three specific bundles: people management, culture, and compliance. By categorizing HRM functions into these three groups, we align with the primary domains where HRM support is most needed in the age of AI integration in the workplace.

Our paper underscores the dynamic evolution of HRM in the era of AI, emphasizing its central role in orchestrating the integrated and symbiotic relationship between humans and machines within organizations. The lack of understanding in implementing AI in a human-centric way highlights the need for a practical approach that goes beyond merely humanizing AI. HRM plays a pivotal role in this area seeing its human-centric focus in the value creation process of organizations and its strategic position within management practice to enhance organizational effectiveness. We propose adopting a multi-disciplinary, human-centric, and integrated approach that can address the current concerns and fears surrounding AI development and deployment in the workplace. AI evolves over a path of maturity spanning a continuum of contemporary cognitive architectures to more socio-cognitive and cross-domain architectures (e.g., Gupta et al., 2023), and in terms of implementation and human-centricity, needs to be interpreted in the context of place and time (Wilkens et al., 2021a). This paper, therefore, categorizes the AI-HRM journey into technocratic, human-AI integration, and fully-embedded AI phases, each presenting unique challenges and opportunities. The benefit of this approach is that it allows organizations to evaluate at which stage of AI implementation and usage they find themselves and the critical role HRM can play in advancing digital transformation efforts and human-AI integration. In our paper, we also anticipate the emergence of advanced human-AI integration paradigms, such as neural integration, emphasizing HRM's role in ensuring ethical, responsible, and fair practices. By looking at the issue from culture, compliance, and people management, our framework not only paves a roadmap toward human-centric AI, but also distinguishes organizations as stewards of technology that enhances, rather than diminishes, the human experience and potential. The paper serves as a forward-looking guide for HRM practitioners, policymakers, and researchers seeking to navigate the transformative landscape of AI in HRM while upholding ethical principles and fostering a future where AI and humans symbiotically co-exist in the workplace.

## Author contributions

AF: Writing—original draft, Writing—review & editing. GM: Writing—original draft, Writing—review & editing. PF: Writing—original draft, Writing—review & editing.
